# Localization Accuracy of Ultrasound-Actuated Needle with Color Doppler Imaging

**DOI:** 10.3390/diagnostics10121020

**Published:** 2020-11-28

**Authors:** Tingyi Jiang, Xinle Zhu, Yang Jiao, Xinze Li, Zhitian Shen, Yaoyao Cui

**Affiliations:** 1 Suzhou Institute of Biomedical Engineering and Technology, Chinese Academy of Sciences, Suzhou 215010, China; zhuxl@sibet.ac.cn (X.Z.); jiaoy@sibet.ac.cn (Y.J.); lixinze@sibet.ac.cn (X.L.); samuelszt@163.com (Z.S.); 2 University of Chinese Academy of Sciences, Beijing 100049, China; 3 Department of Electronic Engineering and Information Science, University of Science and Technology of China, Hefei 230031, China

**Keywords:** color Doppler, needle, localization accuracy, regional anaesthesia, biopsy, ultrasound guidance

## Abstract

An ultrasonic needle-actuating device for tissue biopsy and regional anaesthesia offers enhanced needle visibility with color Doppler imaging. However, its specific performance is not yet fully determined. This work investigated the influence on needle visibility of the insertion angle and drive voltage, as well as determined the accuracy and agreement of needle tip localization by comparing color Doppler measurements with paired photographic and B-mode ultrasound measurements. Needle tip accuracy measurements in a gelatin phantom gave a regression trend, where the slope of trend is 0.8808; coefficient of determination (R^2^) is 0.8877; bias is −0.50 mm; and the 95% limits of agreement are from −1.31 to 0.31 mm when comparing color Doppler with photographic measurements. When comparing the color Doppler with B-mode ultrasound measurements, the slope of the regression trend is 1.0179; R^2^ is 0.9651; bias is −0.16 mm; and the 95% limits of agreement are from −1.935 to 1.605 mm. The results demonstrate the accuracy of this technique and its potential for application to biopsy and ultrasound guided regional anaesthesia.

## 1. Introduction

The ultrasound guidance of needle-based regional anaesthesia and tissue biopsy is widely used clinically [1,2]. Ultrasound imaging visualizes anatomical targets and needles and allows anaesthesiologists, radiologists, and surgeons to place needle tips with precision rather than depend on the palpation of tissues [3]. The ultrasound guidance has improved the efficacy and reduced some side effects during regional nerve block [4]. However, the presentation and interpretation of real-time ultrasound guided needle interventions remain inaccurate and unreliable [5,6,7,8]. Recognition of the tip of the needle is particularly difficult at steep in-plane insertion angles and when targeting deep tissue [4]. An inaccurate needle placement increases the risk of adverse results, such as inadequate anaesthesia, bleeding and nerve damage during regional anaesthesia, as well as mis-sampling and misdiagnosis during biopsy [9,10].

The risks caused by the inaccurate needle placement have motivated a lot of research. Advanced imaging technologies such as beam steering [11,12], image compounding [13], and 3D imaging [14] have been proposed to improve imaging quality. Mechanical guidance [15] and optical guidance [16] have been utilized to optimize the needle-beam-alignment. Echogenic needles were designed and fabricated to increase the intensity of backscattering ultrasound echoes [10]. All of these technologies have shown specific advantages to improve needle visibility, but they all also suffer from limitations and require further development. Another promising method to improve needle visibility is the active needle enhanced imaging technology. Color Doppler imaging can visualize a vibrating needle within a stationary media [14,17]. The benefits of utilizing color Doppler imaging with the ColorMark device (ColorMark, EchoCath Inc., Princeton, NJ, USA) in percutaneous needle procedures were reported in the studies of Feld and Jones [18,19,20]. They reported that color Doppler generated a color image of a vibrating biopsy needle and its tip, as well as improved confidence in detecting a needle, compared with real-time B-mode imaging. However, due to the flexural vibration of ColorMark device, the Doppler image of needle expands into the neighboring tissue, resulting in the difficulty of determining the needle tip position accurately. Moreover, a high-pitched sound was heard due to the vibration at audio frequencies [18].

To further improve the performance of this kind of technique, Sadiq (patent GB 1304798.0, 2013) developed a piezoelectric transducer based on a mass-spring design to actuate a standard needle at low ultrasonic frequency [21]. No high-pitched sound is heard due to the ultrasonic vibration. Simulation and characterization of the ultrasound-actuated needle have confirmed the longitudinal vibration mode and confirmed the improved needle visibility under Doppler imaging [22]. Not only was the needle visibility enhanced, but also the penetration force and needle deflection were reduced [23,24].

The ultrasound-actuated needle with color Doppler imaging is a promising solution for poor needle visibility, but its specific performance is not yet fully determined, for instance the variation of needle visibility caused by the insertion angle. The localization accuracy and agreement data of the actuated needle have not been gathered. Therefore, the primary aim of this work was to investigate the influence on needle visibility of the insertion angle and drive voltage, as well as to determine the needle tip accuracy under color Doppler guidance.

## 2. Methods

### 2.1. Fabrication and Characterization of Needle Actuation Transducer

**Figure 1:**
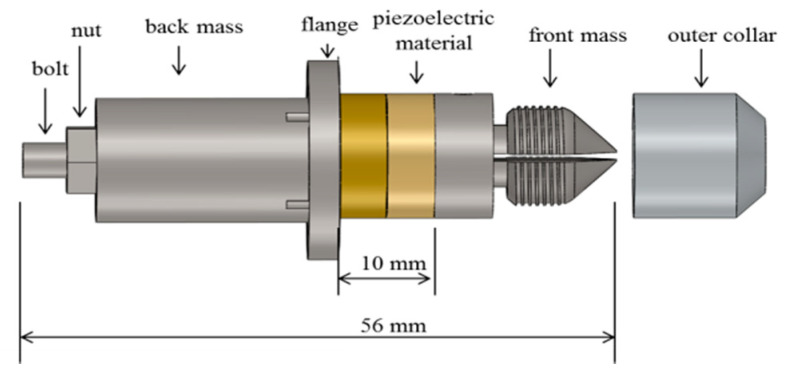
Schematic CAD (solidworks 2017, Dassault Systèmes SA) model of Langevin design needle actuation transducer.

A needle actuation transducer based on the conventional Langevin design was fabricated, as shown in Figure 1. The piezoelectric elements consist of two PZT4 rings (outer diameter 15 mm; inner diameter 8 mm; and thickness 5 mm) (Beijing Ultrasonic, Beijing, China), and are held between the front mass and the back mass with a bolt. The piezoelectric elements are polarized in opposite directions and electrically connected in parallel. Pre-stress is applied to prevent dynamic tensile forces inside the piezoelectric rings. The flange is located at the position of the nodal plane and is used as support for device packaging. An axial opening is created inside the bolt and the front mass to allow the needle to pass through. The needle is clamped to the transducer by tightening the outer collar and oscillates longitudinally when the piezoelectric transducer is excited. After fabrication, a complete characterization was carried out using small and large signal characterization techniques to test the basic and functional performance. Characterization details and results can be found in the previous work [25].

### 2.2. Overall Experimental Setup

**Figure 2:**
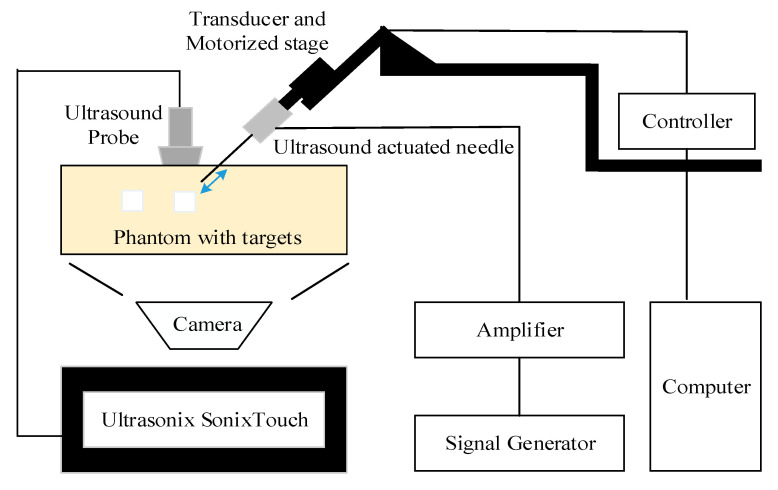
Diagram of experimental setup for the needle accuracy test.

The overall experimental setup was shown in Figure 2. A standard 21 g nerve block needle (TuoRen, Henan, China) was attached to the needle actuation transducer and installed onto the motorized translation stage (MF10, Motorman Robot Co. Ltd., Shenzhen, China). The needle actuation transducer was fabricated in the lab and was actuated at 21.1 kHz with a maximum tip displacement up to 8 µm [25]. The drive signal, provided by a signal generator (DG1062Z, RIGOL TECHNOLOGIES, INC), amplified by a wideband amplifier (ATA-122D, Aigtek, Xian, China), was sinusoidal and kept constant during each insertion. The motorized stage, controlled by a servo controller (CL-01A, Motorman Robot Co. Ltd., China), can be operated manually or through the software to an accuracy of 0.1 mm. The needle was aimed at the target in the phantom or tissue before each insertion. Ultrasound images were acquired using a commercial medical ultrasound imaging scanner (SonixTouch, Ultrasonix, Richmond, Canada) with a wideband of 5–14 MHz linear probe (L14-5/38). The probe was aligned with the plane of needle insertion (“in-plane” configuration). The ultrasound gel was applied for better coupling to the phantom or tissue.

Two studies, the needle visibility and needle accuracy tests, were performed with a similar experimental setup, investigating the visibility and accuracy, respectively.

### 2.3. Needle Visibility Test

In this test, the effect of various factors, including the insertion angle and drive voltage on needle visibility were investigated. The actuated standard needle was inserted into a gelatin phantom and fresh porcine tissue to compare the needle visibility under the B-mode and color mode. Afterwards, the obtained group images were compared and analyzed. The needle actuation transducer was driven at its resonant mode (21.1 kHz) with different drive voltages (0, 5, 15, 25, 35, 45 V) for each angle. The needle was oriented at different angles (30, 45, 60, 70°), before insertion, and gradually advanced to the maximum depth in a 5 mm step by the motorized stage. Here, the static needle (0 V drive voltage) was imaged using the B-mode, representing the standard needle visualized by the B-mode. Gelatin phantom (20%) and fresh porcine tissue were used as specimens for image comparison. The parameter settings for the SonixTouch, as detailed in Table 1, were optimized at the beginning of the test and remained unchanged throughout the test.

### 2.4. Needle Accuracy Test: Color Doppler vs. Photographic

To measure the accuracy of needle tip location under color Doppler guidance, a comparative study was performed in a transparent phantom. The needle was inserted into a transparent gelatin phantom contained in a box with a Perspex side window. Rectangular rubber targets (30 × 15 × 8 mm^3^) were embedded in the phantom at various locations. The rectangular rubber targets were chosen to make it easier to measure the distance from the needle to the target. The possible disadvantages of their material and size are discussed later. A high resolution digital camera was placed in front of the phantom and focused on the plane of the needle tip and target. A tripod equipped with a level calibrator was used to hold the camera and align with the phantom. Repeat experiments were conducted under different insertion angles (30°, 45°, 60°), exploring the relationship between localization errors and the insertion angle.

**Figure 3:**
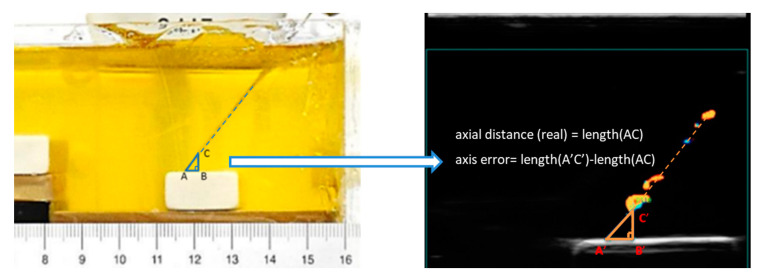
Paired measurements of the distance from the needle tip to the rubber target in the photograph and Doppler ultrasound image. Side AC of the right triangle ABC in the photograph is the real axial distance, the axis error equals the difference between length (A’C’) and length (AC).

During this experiment, the needle was advanced incrementally (initially in 2 mm, then in 0.5 mm steps) towards the target. Paired images were collected at each position of the needle by the ultrasound and photography. Paired measurements of the distance from the needle tip to the rubber target were obtained. The paired images were transferred to a computer for analysis using ImageJ. As shown in Figure 3, we set the intersection point between the straight line where the puncture needle is located and the outer edge of the target as point A. Point C is set as the intersection point between this straight line and the outer edge of the needle tip. To form a right triangle ABC, a point B was set on the target surface. Then, the side AC of the right triangle ABC is the axial distance, BC is the vertical distance, and AB is the horizontal distance. Here, side AC of the right triangle ABC in the photograph is considered to be the real axial distance. According to the above conditions, the accuracy of the needle tip localization is calculated as the axis error, which equals the difference between length A’C’ and length AC. Afterwards, a linear regression analysis was performed. Paired measurements were also compared using the Bland-Altman method to determine the limits of agreement.

The difference between paired measurements (y-axis) was plotted against the mean of each data pair (x-axis) [26,27]. The bias was plotted as the mean difference with the 95% limits of agreement (2SD). The limits of agreement were calculated for dependent, repeated data [28].

### 2.5. Needle Accuracy Test: Color Doppler vs. Greyscale

In this experiment, a gelatin phantom was made incorporating grapes. Grapes are soft with a relatively stiff outer surface and are acoustically and mechanically similar to tumors. Grape phantoms are commonly used in biopsy training [29,30]. Due to the curvature of the grape surface, it was not possible to determine the distance accurately using the photographic method. This is due to the fact that when the plane of the puncture needle is not aligned with the plane of the largest cross-section of the grape, the photographic method will no longer be suitable for a distance measurement. Therefore, the “real” value of the distance in this experiment is unknown. Instead, conventional B-mode imaging was used to visualize the needle tip and from that to measure the distance between the needle tip and grape. The phantom we used had a low density of scatterers. Although it does not give a very realistic image, this is an advantage in this case, as we can see the needle shaft and needle tip in the B-mode rather easier and clearer than in the tissue and allowed us to compare the accuracy between the Doppler and greyscale. The experimental setup was similar to previous one, with the needle advanced incrementally at different angles using a mechanical stage. Paired images were captured from the Doppler mode and B-mode images. A similar linear regression analysis was performed after this experiment and the accuracy was regarded as the mean difference between the tip position on Doppler and B-mode images.

## 3. Results

### 3.1. Needle Visibility Test: Effect of Drive Voltage on Needle Visibility

**Figure 4:**
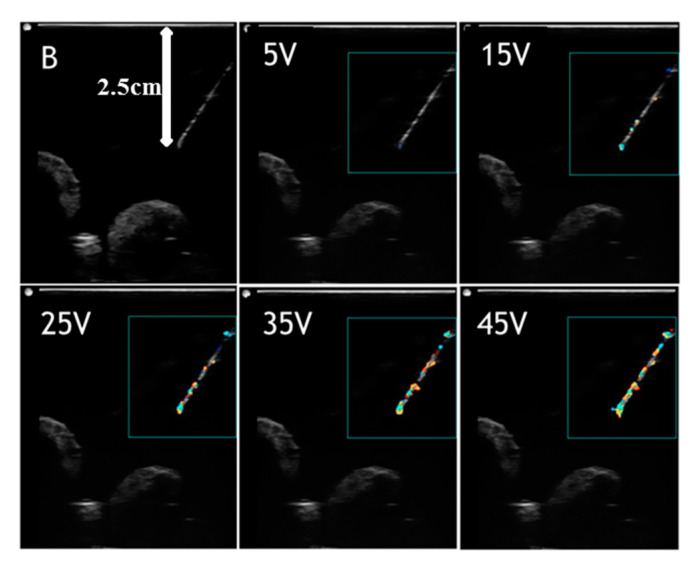
Visualized vibrating needle in gelatin phantom at an angle of 60° and a depth of 2.5 cm with voltages (5, 15, 25, 35, 45 V).

**Figure 5:**
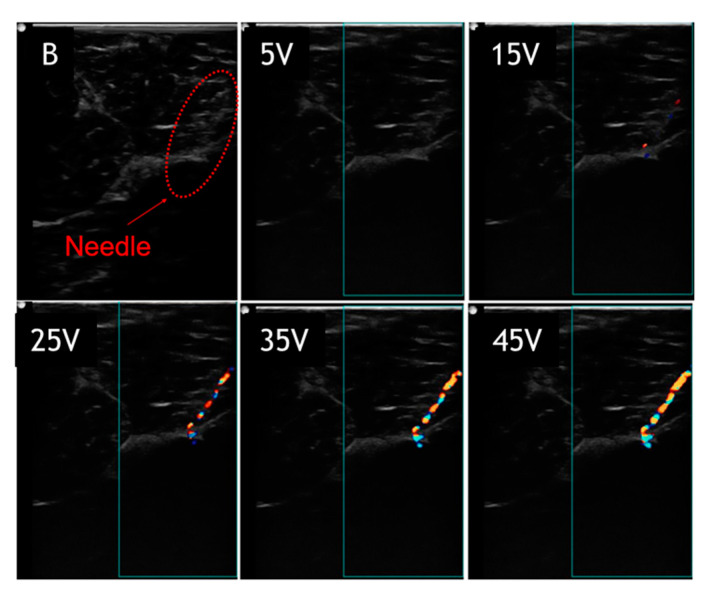
Visualized vibrating needle in porcine tissue at an angle of 60° and a depth of 2.5 cm with voltages (5, 15, 25, 35, 45 V).

Figure 4 and Figure 5 show images of the needle in gelatin phantom and porcine tissue, respectively at 60° and a depth of 2.5 cm. The needle in different images was driven with voltages of 0, 5, 15, 25, 35, and 45 V, respectively. Again, the static needle (0 V drive voltage) was imaged using the B-mode, representing the standard needle visualized by the B-mode. The results indicate that with higher drive voltages, the needle image is better covered with a color pixel from the Doppler mode. In the gelatin phantom (Figure 4), the needle image under the B-mode is quite good, showing the complete needle shaft and distinct needle tip. The needle image under the B-mode is totally blurred in porcine tissue (Figure 5), showing that the needle tip is hard to distinguish and the needle shaft is buried in the background. The actuated needle with higher voltages (25, 35, 45 V) is more visible in the color Doppler mode, especially in the porcine tissue, and the needle tip can be easily localized. This is due to the fact that the needle is highlighted with the needle shaft and the tip is illuminated by the color pixel, making it much more distinguishable.

### 3.2. Needle Visibility Test: Effect of Insertion Angle on Needle Visibility

**Figure 6:**
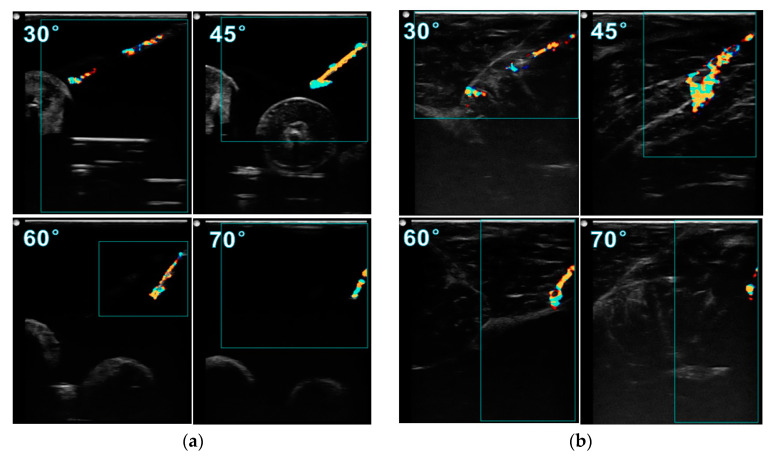
Visualized vibrating needle in (**a**) gelatin phantom and (**b**) porcine tissue with a voltage of 45 V and a depth of 2 cm at insertion angles of 30, 45, 60, 70°.

To investigate how the insertion angle influences the needle visibility, the needle images at insertion angles of 30°, 45°, 60°, and 70° are compared in Figure 6. All the images were obtained when the needle was inserted to a depth of 2 cm and actuated at 45 V. Comparing the images in gelatin phantom, at 45° the image has a better needle visibility than other angles, with the complete needle shaft, distinct needle tip, and great sharpness of the needle surface. Although inserting at 45° provides a good needle image, the artifact at the tip is more obvious than at other angles. This phenomenon is more serious in porcine tissue, as shown in Figure 6b. The tail-shape artifact expands to a large colored area, much greater than the diameter of the needle shaft, making it difficult to localize the tip. It was found that the size of this artifact can be reduced by decreasing the drive voltage. In porcine tissue, it is observed that inserting at 60° gives a better needle image than at 45°, with much less noise at the tip and the complete needle shaft being visible.

### 3.3. Needle Accuracy Test: Color Doppler vs. Photographic

**Figure 7:**
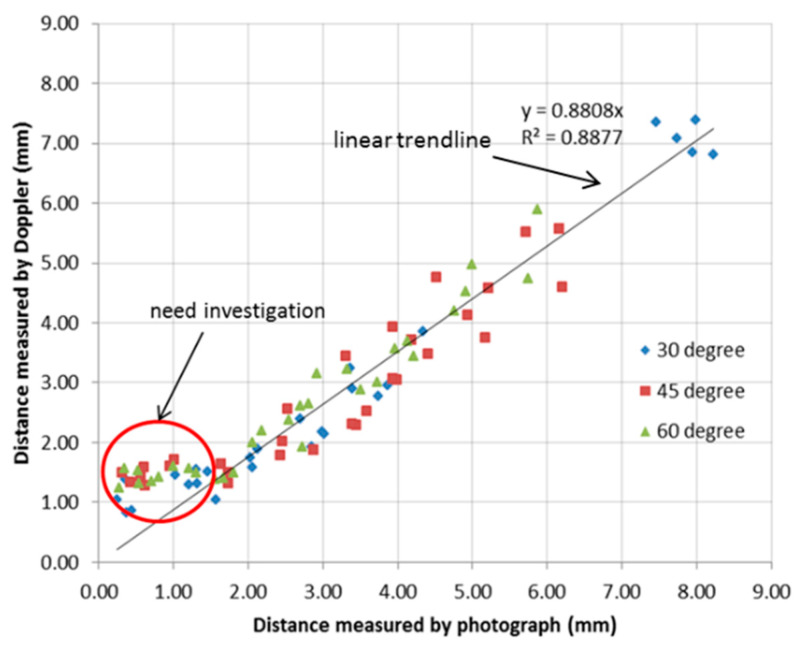
Linear regression analysis of the paired Doppler and photographic measurements.

All the paired measurements of the distance from the needle tip to the rubber target were obtained from the paired ultrasound images and photographs and were plotted as data points in a single diagram. The linear regression analysis was performed in Microsoft Excel (2010) to determine the relationship between the two measurement techniques. Figure 7 shows the relationship between Doppler and photographic measurements. Each point in the graph is calculated from paired images. In the plot, the x-axis is the axial distance measured from the photograph and the y-axis is the axial distance (line AC) measured from the Doppler image. A linear trendline was plotted to fit the data points and the slope was calculated. Here, the distance measured from the photograph was considered to be the “real” distance between the needle tip and target. The slope of the fitted trendline was calculated as 0.8808, indicating that the distance measured from the Doppler image is a little less than the real distance. This is due to the movement of the tip and surrounding tissue, which are both imaged by the Doppler. The size of the needle tip displayed in the Doppler image is normally larger than its real size, consequently reducing the estimated distance between the tip and target.

However, the distance measured from the Doppler image is found to be much larger than the real distance when the distance is less than 1.5 mm. These anomalous data points are marked by the red ring and require further investigation.

**Figure 8:**
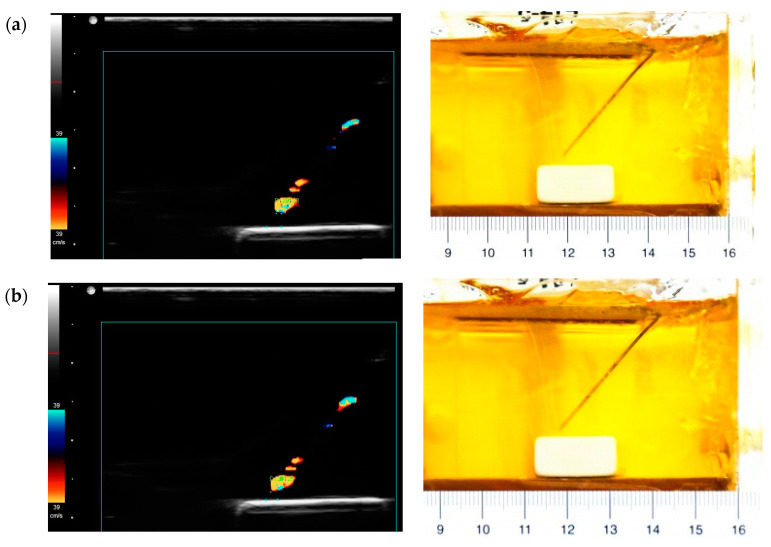

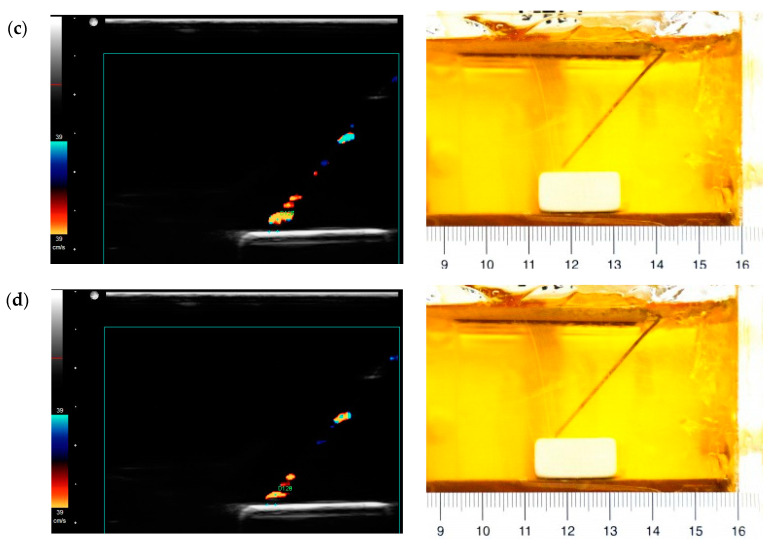
Paired images obtained from the Doppler ultrasound (**left**) and photographs (**right**) when the distance is about (**a**) 1.5, (**b**) 1, (**c**) 0.5, (**d**) 0 mm.

In order to investigate these anomalous data points, images showing the needle tip close to the target were studied. The images in Figure 8 show the position where the needle tip is about 1.5, 1, 0.5 mm away from the target. The needle tip is actually moving close to the target, as can be seen in the photographs, but the distance measured from the corresponding Doppler image does not match. It is observed that the color area at the tip gradually reduces as it approaches the target. Based on the principle of color Doppler imaging, the Doppler signal is capturing the movement of the needle and surrounding media. Here, a high rubber-gelatin bonding strength could dampen the vibration at the rubber-gelatin interface, making the color area at the tip smaller. It also indicates that the rubber target could cause measurement errors at final positions.

Due to the above problems and observations close to the rubber target, the data points with a distance ≤1.5 mm, were excluded from the final analysis. About 30 paired measurements for each angle were used for the final calculation of average errors and standard deviation. Table 2 summarizes the needle tip localization error determined for each insertion angle. Here, the average errors for all different angles are negative, meaning that the measured distance in the Doppler image is in general smaller than the real distance. The 60° needle insertion has a smaller error than the other insertion angles, which can be attributed to fewer artifacts observed at this angle.

**Figure 9:**
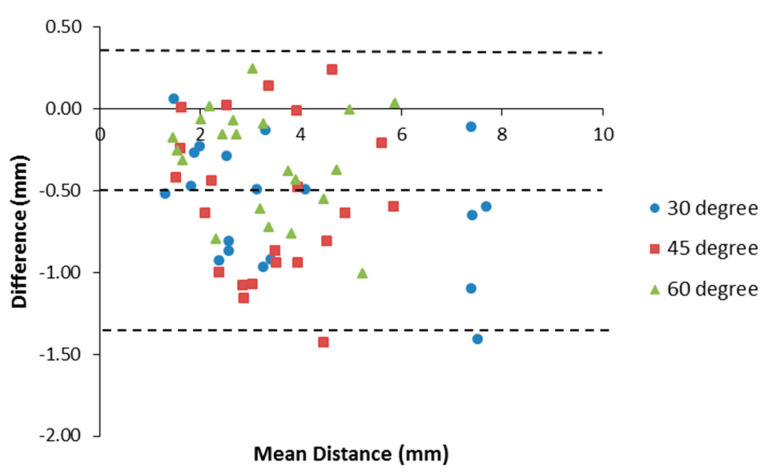
Bland-Altman plot of the difference between Doppler and photographic measurements against the mean of the distance from both methods.

Furthermore, by applying the Bland-Altman method, a Bland-Altman plot showed an agreement between Doppler and photographic measurements, as shown in Figure 9. The bias was −0.50 mm and the 95% limits of agreement were from −1.31 to 0.31 mm, containing 95% (67/69) of the difference data points.

### 3.4. Needle Accuracy Test: Color Doppler vs. Greyscale

**Figure 10:**
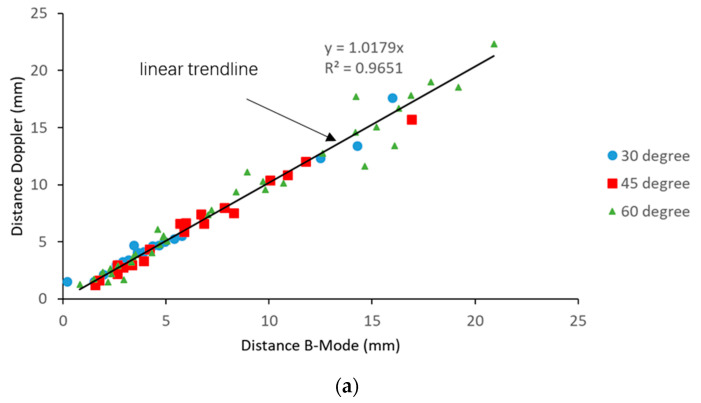

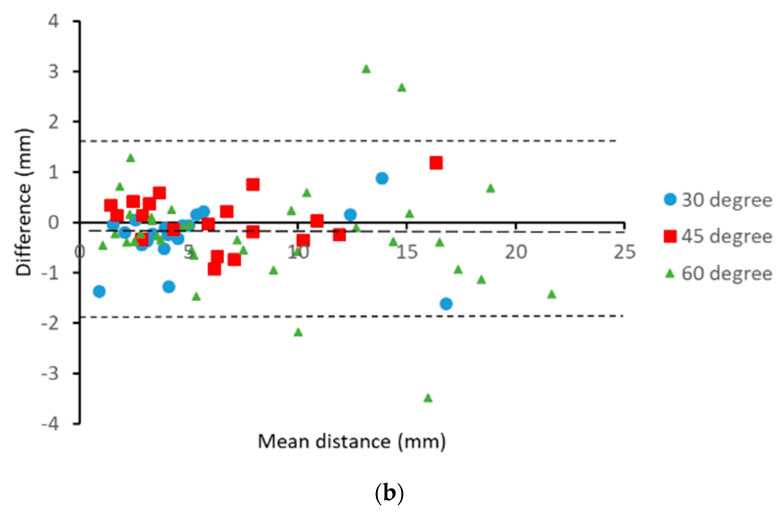
(**a**) Relationship between the B-mode and Doppler measurements and (**b**) Bland-Altman plot of the difference between the Doppler and B-mode measurements against the mean of the distance from both modalities.

Paired images were captured from the color Doppler mode and B-mode. The distances between the needle tip and the grape target were measured for each modality. The relationship between the B-mode and Doppler mode measurements is plotted in Figure 10a. The trendline through these scatter points was plotted and the coefficient of determination was calculated as 0.9651, indicating a good fit of data points. The slope of the trendline was 1.0179, very close to 1, indicating a close agreement between the two modalities. As discussed above, the needle tip accuracy with the rubber target is only valid for a distance ≥ 1.5 mm, from the target. In this test, no anomalous data points were observed when the needle tip was close to the grape target. It is possibly due to the fact that the grapes eliminate the vibration absorption phenomenon, and consequently overcome this limitation.

A Bland-Altman plot showed an agreement between the B-mode and Doppler mode measurements, as shown in Figure 10b. The bias was −0.16 mm and the 95% limits of agreement were from −1.935 to 1.605 mm, containing 95% (70/74) of the difference data points, which means that the color Doppler method agrees sufficiently well with the B-mode. In general, data points were evenly distributed on both sides of the mean line, indicating a uniform difference along the insertion path.

## 4. Discussion

In this paper, we investigated the influence on needle visibility of the insertion angle and drive voltage and also determined the accuracy and agreement of needle tip localization by comparing color Doppler measurements with paired photographic and B-mode ultrasound measurements. Experiments using rubber inclusions demonstrated the accuracy and agreement of Doppler imaging to the photographic measurement at a distance > 1.5 mm (the slope of trend line is 0.8808; coefficient of determination (R^2^) is 0.8877; bias is −0.50 mm; and the 95% limits of agreement are from −1.31 to 0.31 mm). All errors are negative with a consistent underestimation of the order of 0.5 mm, creating a safety margin. The accuracy was maintained at high angulations (Table 2). As the visibility of the needle tip and shaft is often lost with high angulations in clinical practice, the actuated needle offers a distinct advantage. When comparing the color Doppler with B-mode ultrasound measurements, the slope of the regression trend is 1.0179, R^2^ is 0.9651, and bias is −0.16 mm, indicating a very good agreement. The 95% limits of agreement are slightly disappointing at almost −2.0 to over 1.5 mm.

There are two possible causes of measurement error in the study, which are artifacts and miscalculation due to the wrong assumption for the speed of sound. Artifacts are misrepresentations of an object in the image. These artifacts are likely caused by the ultrasound reflection and reverberation from the bevel tip of the needle. According to our observation, these artifacts occur at both the B-mode and Doppler mode. In addition, it is found that the needle tip in the Doppler image is larger than the real needle. This is due to the fact that much of the detected Doppler signal comes from the stronger echoes from the tissue or tissue mimic adjacent to the needle as it is dragged back and forth by the friction of the needle. Therefore, the apparent tip is extended slightly reducing the measured distance to the target. This can at least partially explain the consistent underestimation of the tip target distance, but it should be stressed that this builds in a small safety margin. An error in the other directions would be unacceptable.

In the needle accuracy test, the rubber targets were chosen since they had flat surfaces and were clearly visible under the ultrasound. They were easily embedded in a gelatin phantom without damage and clear enough to help judge the moment of contact. However, our experiments gave inaccurate results at distances < 1.5 mm from the target. It is thought that the high rubber-gelatin bonding strength would weaken the vibration at the rubber-gelatin interface, resulting in a smaller color zone at the tip. Using the grape target, no similar aberration was observed.

In this study, the speed of the ultrasound is set by the SonixTouch system to be 1540 ms^−1^. Othman reported that the speed of the ultrasound in gelatin varies with time from 1390 to 1500 ms^−1^ [31]. However, the phantom we used was fairly freshly made and the sound speed was measured to be about 1410 ms^−1^ using the time of flight method. This could lead to an error in depth calculation of 8%. However, due to the relative angle between the needle and the imaging transducer, the errors will be less. Here, a linear probe was used and the target and needle tip were visualized in the field of view. Therefore, the lateral distance may be assumed to be correctly measured and the error is mainly in the depth orientation. The resultant error in estimation of axial distance due to the error of the ultrasound speed at worst will be 2% at 30°, 4% at 45°, and 6% at 60°. This effect may contribute to the regression coefficient being less than one but cannot explain the full extent. This problem only affects the comparison between the Doppler and photographic measurements. The error due to the speed of sound affects the Doppler and greyscale to the same extent and therefore, has no influence on that comparison.

Motion artifacts can occur, but in general, these appear with the possible geometric envelope of the needle and can generally be disregarded, although they may contribute to some of the imprecise measurements in the second part of the work, where the artifacts appear around the needle tip. If the needle is not absolutely in the imaging plane, it is possible that part of the needle shaft image is missing. This is most serious if the tip is included and care must be taken during insertion in order for the tip to be consistently visualized during its progression. Several paired data points in the second part showed large differences ≥ 3 mm. It is possible that the needle was not perfectly aligned with the ultrasound imaging plane, leading to the invisibility of the needle tip, in these cases, the end of the visible shaft was considered as the needle tip.

In clinical practice, the required accuracy of the needle intervention varies depending on the application. For example, the desired performance in some common needle procedures such as biopsies for prostate, kidney, breast, and liver is the millimeter level, while in brain, foetus, eye, and ear the placement accuracy of micrometers is desirable [32]. In a recent study, a very promising technique, known as the passive magnetic tracking needle guidance technology (NGT), was reported to have a placement accuracy of 2 mm better than the conventional ultrasound [33]. In comparison, the results indicate an accuracy of 1.310 mm in color Doppler vs. the photographic experiment and an accuracy of 1.935 mm in color Doppler vs. the grayscale experiment. Our results support the potential of the ultrasound-actuated needle in a biopsy in the prostate, kidney, breast, and liver. In addition, the advantages of the actuated Doppler needle, including enhanced visibility of the needle and reduced penetration force, can both be utilized during needle insertion [22,23].

Further studies are warranted investigating the accuracy and reliability of this device in animal and cadaver tissue. It would be interesting to repeat the photographic comparison using smaller, more compliant targets and in a tissue mimic doped for the correct speed of sound. The accuracy should also be tested in an appropriately hydrated tissue. For regional anaesthesia, the actuated needle may have enough accuracy for an ultrasound guided nerve block of superficial structures, which have well delineated epineurium and marked acoustic contrast to the surrounding tissue. Realistically, we feel that the 95% limits of agreement should be contained between −1 and +1 mm for regional anaesthesia and it is our intention to work towards that goal with a focus on the artifact reduction algorithm.

## Figures and Tables

**Table 1:** Parameter settings of the SonixTouch ultrasound system.

Parameter	20% Gelatin Phantom	Porcine Tissue
Greyscale frequency	10.0 MHz	10.0 MHz
Doppler frequency	6.6 MHz	4.0 MHz
Depth	5.0 cm	5.0 cm
Sector	100%	100%
Greyscale gain	55%	60%
Doppler gain	50%	60%
Frame rate	8 Hz	7 Hz
PRF /WF	4 kHz/1440 Hz	4 kHz/840 Hz
Assumed speed of ultrasound	1540 ms^−1^	1540 ms^−1^

**Table 2:** Needle tip localization error determined by the axial distance comparison between Doppler and photographic measurements.

Insertion Angle	Total Number of Paired Measurements	Number of Excluded Measurement	Average Axis Error (mm)	Standard Deviation (SD) (mm)
30°	27	7	−0.59	0.38
45°	37	9	−0.58	0.44
60°	30	9	−0.32	0.32
All angles	94	25	−0.50	0.41

## References

[1] Baloch Z.W., Tam D., Langer J., Mandel S., LiVolsi V.A., Gupta P.K. (2000). Ultrasound-guided fine-needle aspiration biopsy of the thyroid: Role of on-site assessment and multiple cytologic preparations. Diagn. Cytopathol..

[2] Marhofer P., Greher M., Kapral S. (2005). Ultrasound guidance in regional anaesthesia. Br. J. Anaesth..

[3] Rha D.-W., Im S.H., Lee S.C., Kim S.-K. (2010). Needle Insertion into the Tibialis Posterior: Ultrasonographic Evaluation of an Anterior Approach. Arch. Phys. Med. Rehabil..

[4] Munirama S., Joy J., Columb M., Habershaw R., Eisma R., Corner G., Cochran S., McLeod G.A. (2014). A randomised, single-blind technical study comparing the ultrasonic visibility of smooth-surfaced and textured needles in a soft embalmed cadaver model. Anaesthesia.

[5] Abolhassani N., Patel R.V. Deflection of a flexible needle during insertion into soft tissue. Proceedings of the 2006 International Conference of the IEEE Engineering in Medicine and Biology Society.

[6] Chin K.J., Perlas A., Chan V.W., Brull R. (2008). Needle Visualization in Ultrasound-Guided Regional Anesthesia: Challenges and Solutions. Reg. Anesthesia Pain Med..

[7] Hussain H.K., Kingston J.E., Domizio P., Norton A.J., Reznek R.H. (2001). Imaging-Guided Core Biopsy for the Diagnosis of Malignant Tumors in Pediatric Patients. Am. J. Roentgenol..

[8] Roberson P.L., Narayana V., McShan D.L., Winfield R.J., McLaughlin P.W. (1997). Source placement error for permanent implant of the prostate. Med. Phys..

[9] Guo S., Schwab A., McLeod G., Corner G., Cochran S., Eisma R., Soames R. (2012). Echogenic Regional Anaesthesia Needles: A Comparison Study in Thiel Cadavers. Ultrasound Med. Biol..

[10] Hebard S., Hocking G. (2011). Echogenic Technology Can Improve Needle Visibility During Ultrasound-Guided Regional Anesthesia. Reg. Anesthesia Pain Med..

[11] Baker J.A., Soo M.S., Mengoni P. (1999). Sonographically guided percutaneous interventions of the breast using a steerable ultrasound beam. Am. J. Roentgenol..

[12] Cheung S., Rohling R. (2004). Enhancement of needle visibility in ultrasound-guided percutaneous procedures. Ultrasound Med. Biol..

[13] Saleh A., Ernst S., Grust A., Furst G., Dall P., Modder U. (2001). Real-time compound imaging: Improved visibility of biopsy needles and localization wires as compared to single-line ultrasonography. Rofo-Fortschritte Auf Dem Gebiet Der Rontgenstrahlen Und Der Bildgebenden Verfahren.

[14] Fronheiser M.P., Wolf P.D., Idriss S.F., Nelson R.C., Lee W., Smith S.W. (2004). Real-Time 3D Color Flow Doppler for Guidance of Vibrating Interventional Devices. Ultrason. Imaging.

[15] Kettenbach J., Kronreif G., Figl M., Birkfellner W., Hanel R., Bergmann H. (2004). Robot-assisted biopsy using ultrasound guidance: Initial results from in vitro tests. Eur. Radiol..

[16] Tsui B.C.H. (2007). Facilitating needle alignment in-plane to an ultrasound beam using a portable laser unit. Reg. Anesthesia Pain Med..

[17] Powers J.E. (1992). Ultrasonic Imaging of Biopsy Needle. U.S. Patent.

[18] Feld R., Needleman L., Goldberg B.B. (1997). Use of needle-vibrating device and color Doppler imaging for sonographically guided invasive procedures. Am. J. Roentgenol..

[19] Jones C.D., McGahan J.P., Clark K.J. (1997). Color Doppler ultrasonographic detection of a vibrating needle system. J. Ultrasound Med..

[20] Armstrong G., Cardon L., Vilkomerson D., Lipson D., Wong J., Leonardorodriguez L., Thomas J.D., Griffin B.P. (2001). Localization of needle tip with color doppler during pericardiocentesis: In vitro validation and initial clinical application†. J. Am. Soc. Echocardiogr..

[21] Sadiq M., Cochran S., Huang Z., Corner G., McLeod G., Carena P. (2014). Medical Apparatus and Its Visualization. U.S. Patent.

[22] Kuang Y., Hilgers A., Sadiq M., Cochran S., Corner G., Huang Z. (2016). Modelling and characterisation of a ultrasound-actuated needle for improved visibility in ultrasound-guided regional anaesthesia and tissue biopsy. Ultrasonics.

[23] Liao X.C., Sadiq M., Corner G., Cochran S., Huang Z.H. (2013). Reduced Penetration Force through Ultrasound Activation of a Standard Needle an Experimental and Computational Study. Proceedings of the 2013 IEEE International Ultrasonics Symposium (IUS).

[24] Sadiq M.R., Cochran S., Liao X., Huang Z. Enhanced US-guided needle intervention through ultrasound actuation of a standard needle. Proceedings of the 2014 IEEE International Ultrasonics Symposium.

[25] Jiang T., Xia C., Cochran S., Huang Z. (2018). Improved Performance of d(31)-Mode Needle-Actuating Transducer With PMN-PT Piezocrystal. IEEE Trans. Ultrason. Ferroelectr. Freq. Control..

[26] Bland J., Altman D. (1995). Comparing methods of measurement: Why plotting difference against standard method is misleading. Lancet.

[27] Bland J.M., Altman D.G. (1999). Measuring agreement in method comparison studies. Stat. Methods Med. Res..

[28] Myles P., Cui J.I. (2007). Using the Bland–Altman method to measure agreement with repeated measures. Br. J. Anaesth..

[29] Bude R.O., Adler R.S. (1995). An easily made, low-cost, tissue-like ultrasound phantom material. J. Clin. Ultrasound.

[30] Fornage B.D. (1989). A simple phantom for training in ultrasound-guided needle biopsy using the freehand technique. J. Ultrasound Med..

[31] Othman N.S., Jaafar M.S., Rahman A.A., Sazlinayati E. Ultrasound propagation speed of polymer gel mimicked human soft tissue in 23 days. Proceedings of the 2011 International Conference on Biomedical Engineering and Technology.

[32] Abolhassani N., Patel R., Moallem M. (2007). Needle insertion into soft tissue: A survey. Med. Eng. Phys..

[33] Johnson A.N., Peiffer J.S., Halmann N., Delaney L., Owen C.A., Hersh J. (2017). Ultrasound-Guided Needle Technique Accuracy: Prospective Comparison of Passive Magnetic Tracking Versus Unassisted Echogenic Needle Localization. Reg. Anesthesia Pain Med..

