# Erratum: Jiang et al. Localization Accuracy of Ultrasound-Actuated Needle with Color Doppler Imaging. *Diagnostics* 2020, *10*, 1020

**DOI:** 10.3390/diagnostics11050755

**Published:** 2021-04-23

**Authors:** Tingyi Jiang, Graeme McLeod, Zhihong Huang, Xinle Zhu, Yang Jiao, Xinze Li, Zhitian Shen, Yaoyao Cui

**Affiliations:** 1 Suzhou Institute of Biomedical Engineering and Technology, Chinese Academy of Sciences, Suzhou 215010, China; zhuxl@sibet.ac.cn (X.Z.); jiaoy@sibet.ac.cn (Y.J.); lixinze@sibet.ac.cn (X.L.); samuelszt@163.com (Z.S.); 2 Institute of Academic Anaesthesia, University of Dundee, Dundee DD1 4HN, UK; g.a.mcleod@dundee.ac.uk; 3 School of Science and Engineering, University of Dundee, Dundee DD1 4HN, UK; z.y.huang@dundee.ac.uk; 4 University of Chinese Academy of Sciences, Beijing 100864, China; 5 Department of Electronic Engineering and Information Science, University of Science and Technology of China, Hefei 230031, China

The authors wish to correct the following erratum in this paper [1].

In the original paper [1], Graeme McLeod and Zhihong Huang were not included as authors in the published article. The corrected author contributions statement appears here. The authors apologize for any inconvenience caused and state that the scientific conclusions are unaffected. The original article has been updated.

## References

[1] Jiang T., Zhu X., Jiao Y., Li X., Shen Z., Cui Y. (2020). Localization Accuracy of Ultrasound-actuated Needle with Color Doppler Imaging. Diagnostics.

